# Factors associated with anxiety, stress, depression and burden among informal caregivers of patients with dementia: a cross-sectional study

**DOI:** 10.1186/s12912-025-04014-8

**Published:** 2025-11-11

**Authors:** Sofia Canedo Garrido, Sónia Teixeira, Gerard Mora-Lopez, Francisco Sampaio

**Affiliations:** 1 Nursing Department, Campus Catalunya, Rovira i Virgili University, 35, Catalunya avenue, 43002 Tarragona, Spain; 2 Integrated Mental Health Responsibility Centre, Local Health Unit of Matosinhos, Rua Dr. Eduardo Torres, 4464-513 Matosinhos, Portugal; 3 RISE-Health, Nursing School of the University of Porto (ESEP), 830, 844, 856, Rua Dr. António Bernardino de Almeida, 4200-072 Porto, Portugal; 4 Fernando Pessoa School of Health (ESS-FP), 334, Rua Delfim Maia, 4200-253 Porto, Portugal; 5 Nursing School of the University of Porto, 830, 844, 856, Rua Dr. António Bernardino de Almeida, 4200-072 Porto, Portugal

**Keywords:** Anxiety, Stress, Depression, Burden, Caregiving, Dementia, Mental health

## Abstract

**Background:**

In Europe, demographic ageing and an increase in the prevalence of dementia are expected. Portugal, along with other Mediterranean and Southeastern European countries, exceeds the European trend placing growing pressure on health and social care systems. Informal caregivers play a crucial role in meeting these demands, despite the impact on their own mental health. The emerging and multifaceted nature of informal caregiving, combined with the limited evidence from Southern European contexts, motivated this study. This research aimed (1) to identify the levels of anxiety, stress, depressive symptoms, and burden among informal caregivers of people with dementia in Portugal, and (2) to identify which sociodemographic and/or clinical factors are associated with anxiety, depression, stress symptoms, and burden in these caregivers.

**Methods:**

A survey was conducted among informal caregivers of non-institutionalized dementia patients. The survey encompassed a sociodemographic and clinical assessment using the Positive Mental Health Questionnaire (PMHQ), the Psychological Vulnerability Scale (PVS), the Caregiver Burden Scale (CBS), and the Depression, Anxiety, and Stress Scale – 21 items (DASS21). Linear regressions were performed using caregivers’ data on depression, anxiety, stress, and burden as dependent variables.

**Results:**

A total of 414 caregivers participated. They exhibited normal intensity of depression, anxiety, and stress symptomatology. Several variables significantly related to these outcomes were identified and quantified. The mean CBS score was 54.3 ± 17.8 (range 22–110) and was associated to caregiver age, years in the caregiver role, hours of daily care, and participation in leisure or relaxation activities. Positive mental health and psychological vulnerability were significant explanatory factors for all assessed outcomes.

**Conclusions:**

Greater time dedicated to caregiving and reduced engagement in leisure or relaxation activities were significantly associated with higher burden. The level of positive mental health is a robust associated factor of mental health issues among caregivers.

## Background

In Europe, demographic ageing is expected to double prevalence of dementia by 2050, from over 21 million cases reported in 2021 [1, 2]. In 2025, the highest prevalence rates, according to the proportion of the population affected, are expected in Italy (2.44%), Greece (2.37%) and Portugal (2.29%) - all Mediterranean and Southeastern European countries - exceeding the overall European average of 2.0%. In Portugal alone, more than 200,000 cases are projected for 2025. This is driven by the increasing proportion of adults aged over 70 years [3–5]. The rising costs associated with dementia care will place greater pressure on informal caregivers, social support services, and primary healthcare systems, while the pool of informal caregivers is expected to stagnate despite their growing recognition [6–9].

Caregiving can yield positive outcomes - such as purpose, gratitude, self-realisation, family cohesion, and personal growth - yet these are often overshadowed by sustained demands [10–13]. Caring for individuals with cancer or dementia imposes greater physical and emotional strain than other caregiving contexts, with dementia-related cognitive decline introducing unique challenges that intensify burden and reduce quality of life. Balancing the needs of the person with dementia and those of the caregiver is particularly difficult and tends to worsen with the presence of neuropsychiatric symptoms, advanced disease stages, and high levels of functional dependence [14–24].

The caregiver burden is defined as “the physical, psychological or emotional, social, and financial problems that can be experienced by family members caring for impaired older adults” [25] and could be explained by the Caregiver Stress Process Model, which conceptualises caregiver burden as resulting from background and contextual factors, distinguishing between primary stressors- direct caregiving demands- and secondary stressors from role and intrapsychic strains. These secondary stressors are critical, as they influence self-concept and increase vulnerability to depression and anxiety, whereas coping strategies and social support acting as mediators [26, 27].

The positive dimension of mental health was consolidated by the World Health Organization in 2001, recognising that “concepts of mental health include subjective well-being, perceived self-efficacy, autonomy, competence, intergenerational dependence, and self-actualization of one’s intellectual and emotional potential, among others” [28].

Building upon Jahoda’s theoretical framework, Lluch (1999) developed the Multifactorial Model of PMH with six key factors: (F1) personal satisfaction; (F2) pro-social attitude; (F3) self-control; (F4) autonomy; (F5) problem solving; and (F6) self-realization and interpersonal skills. Strengthening these dimensions fosters optimal human functioning. Higher PMH is linked to improved outcomes [29–31]. There is evidence that PMH promotion programmes can reduce caregiver burden. Therefore, the assessment of PMH among informal caregivers of people with dementia should be considered [32–35]. Psychological vulnerability is negatively associated with PMH and is defined as “a pattern of cognitive beliefs reflecting a dependence on achievement or external sources of affirmation for one’s sense of self-worth”. Such dependence on external achievements and others for self-affirmation, rather than on one’s inner qualities and character, makes an individual’s sense of self-worth vulnerable to others’ reactions and to life’s uncertainties [36, 37]. Within this context, nurses play a central role in dementia care, working collaboratively with caregivers to foster supportive environments and implement non-pharmacological strategies to maintain the caregivers engaged in care provision while protecting their mental health [38–41].

Research has focused on factors related to the person with dementia which have been identified as key determinants of caregivers’ health and perceived burden. However, fewer studies have examined the characteristics and mental health of the caregivers themselves [42, 43]. This study focused exclusively on informal caregivers and factors were based on national and international evidence linking them to depression, anxiety, stress, and caregiver burden. These factors included sociodemographic and caregiving variables: age, sex, marital status, education, employment, caregiver–recipient relationship, caregiving duration, sole caregiving, daily care hours, residential distance, and psychiatric diagnosis [7, 20, 42–49]. Self-care strategies were also considered, including sleep satisfaction, physical activity, healthy diet, leisure and relaxation, and social engagement [50–53].The multifactorial nature of caregiver burden complicates the identification of determinants of stress, anxiety, depression, and burden which are influenced healthcare and welfare systems. Moreover, the predominance of studies in Asian populations limits the transferability of findings to Western contexts [54–56]. This study, conducted in Portugal, expands knowledge in a Southern European context where dementia prevalence is high, intergenerational care norms place substantial pressure on families, and co-residential caregiving - typically recognised as a more intense form of care - is common [49, 57–60]. In addition to complementing existing literature, it addresses two underexplored constructs - PMH and psychological vulnerability. Further research is required to deepen the understanding of key associated factors and to guide the development of targeted, context-sensitive interventions. This research aimed to (1) identify the levels of anxiety, stress, depressive symptoms, and the burden among informal caregivers of people with dementia in Portugal and (2) to identify which sociodemographic and/or clinical factors are determinants of anxiety, depression, stress symptoms and the burden expressed by the caregivers.

## Methods

### Design

This cross-sectional study follows the STROBE guidelines’ recommendations [61] and adheres to the Declaration of Helsinki. Ethical approval was obtained from the Local Health Unit of Matosinhos (40/CES/JAS) and Rovira i Virgili University (OCAS-717) in April 2023. All respondents provided informed consent.

### Participants and setting

The study participants were informal caregivers of non-institutionalized dementia patients, in Portugal without regional restrictions. A convenience sample was recruited through snowball sampling via five strategies: (1) direct invitation through health professionals; (2) social services; (3) the Alzheimer Portugal Association; (4) caregivers’ associations; and (5) self-enrolment through social media. The eligibility criteria for participants were: (1) be an informal caregiver of non-institutionalized dementia patients; (2) be over 18 years old; (3) have contact with the person for at least one hour per week; and (4) be able to read and write in Portuguese. A minimum threshold of one hour per week was adopted, as it ensures that participants had a meaningful level of involvement in caregiving activities while also maintaining comparability with previous studies that have operationalised caregiving intensity using similar metrics [9, 23]. The data were collected from July 2023 to February 2024 through anonymous self-reported pen-and-paper or digital questionnaires (via Microsoft 365) on the basis of preferences and digital literacy. To participate, individuals confirmed that they met al.l the eligibility requirements in both formats.

A minimum sample size of 384 participants was considered necessary for a margin of error of 5%, a 95% confidence interval, and an estimated 50% response distribution (the sample size was calculated by Raosoft, 2024).

### Measurements

The questionnaire in this survey collected the caregiver’s sociodemographic variables: age, sex, level of education, marital status, information on caregiving history (years as a caregiver, formal or informal support, hours per day caring), mental health status, and health-promoting behaviours of participants (healthy nutrition, social activity outside the context of caring, practicing relaxing, leisure, or physical activities). The data collection instruments used included the Positive Mental Health Questionnaire (PMHQ) [62], the Psychological Vulnerability Scale (PVS) [36], the Caregiver Burden Scale (CBS) [63], and the Depression, Anxiety, and Stress Scale-21 (DASS-21) [64], with an overall estimated completion time of 15 min. The questionnaire did not include embedded data quality checks such as attention questions. However, was applied screening criteria at the recruitment stage and were excluded incomplete responses during data cleaning.

### Positive mental health questionnaire (PMHQ)

The PMHQ was conceptualised by Lluch-Canut in Spain [65, 66] and translated and validated for Portugal [62]. This instrument consists of 39 items that assess thoughts, feelings, and behaviours, grounded in the multifactorial model of Positive Mental Health and its six key factors. The participants were invited to select the frequency option they most identified with, presented on a 4-point Likert scale (1 to 4): “always or almost always” (1), “most of the time” (2), “sometimes” (3), and “rarely or never” (4). This instrument makes it possible to calculate the level of positive mental health for each factor, allowing the identification of which factor, or factors, requires intervention [31, 33, 34]. The score ranges from 39 to 156, with the possibility of categorizing into different levels depending on the results, namely low or languishing (39 to 78), intermediate (79 to 117), high or flourishing (118 to 156), with a higher score representing higher level of Positive Mental Health. The PMHQ demonstrated excellent internal consistency (Cronbach’s alpha = 0.92). The internal consistency of each factor ranged from 0.60 to 0.84, and the scale presented good test-retest reliability (0.98). Within this theoretical framework, the isolated analysis of each key factor provides limited interpretative value and was therefore not conducted separately [62].

### Psychological vulnerability scale (PVS)

The PVS was designed and validated by Sinclair and Wallston in the United States and applied to chronically ill adults to identify vulnerable individuals. The Cronbach’s alpha coefficient ranged from 0.71 to 0.87 [37]. Nogueira and collaborators (2017) translated and adapted this instrument to European Portuguese in a sample of higher education students. This self-reported six-item instrument was designed to screen harmful cognitive patterns related to perceptions of social dependence, negative attributions, self-oriented perfectionism, and reliance on external sources of approval [35]. The participants are invited to assess each statement with a 5-point Likert scale (1 to 5), from (1) = “does not describe me at all” to (5) = “describes me very well”. The higher the score is, the greater the degree of psychological vulnerability, which may range from 6 to 30. The PVS demonstrated good validity, reliability, and stability. The Cronbach’s alpha coefficient was 0.73 [35].

### Depression anxiety stress scale-21 (DASS-21)

The Depression Anxiety Stress Scale with 21 items (DASS-21) is the short version of the original DASS, which is composed of 42 items, and was developed by Lovibond and Lovibond (1995) with Australian university students. This self-reported tool represents a three-part model that assesses emotional states with three subscales: depression, anxiety, and stress. This instrument is widely used when a brief screening is needed [67]. This study used the Portuguese version with internal consistencies of 0.85 (depression), 0.74 (anxiety), and 0.81 (stress) for each subscale [64]. Each item consists of a sentence listing negative emotional symptoms. The participants are invited to assess each statement with a 4-point Likert scale (0 to 3), from (0) = “nothing applied to me” to (3) = “applied to me most of the time”, indicating the extent to which they experienced each symptom in the last week. The scale provides three scores, one per subscale, ranging from 0 to a maximum of 21. Higher scores represent more negative affective states [64, 67].

### Caregiver burden scale (CBS)

The first version of the Zarit Caregiver Burden Scale was developed in the United States of America by Steven Zarit and collaborators and was administered to primary caregivers of persons with Dementia [68]. This self-report instrument measures objective and subjective burden, providing useful information on the impact of caregiving in areas such as health, social and personal life, financial condition, emotional well-being, perception of self-efficacy, and interpersonal relationships, as reported by caregivers [63, 68, 69]. The Portuguese version was chosen for this research, with a Cronbach’s alpha of 0.93 [63]. Each item is scored from (1) = “never” to a maximum of (5) = “nearly always”. This scale presents a score range from 22 to 110, where a higher score corresponds to a greater perceived burden, with the following cut-off points: no burden (22–46 points), mild burden (47–55 points), and severe burden (56–110 points) [63].

### Statistical analysis

The statistical analysis was performed via IBM SPSS version 29. The data analysis tests were considered significant for *p*-values equal to or less than 0.05.

The sociodemographic attributes of the sample were quantitatively assessed through central tendency and dispersion indices for quantitative variables, alongside absolute and relative frequencies for qualitative variables. Furthermore, the evaluation of outcome variables, encompassing individual factors and aggregate scale scores, utilized mean values and standard deviations (SD). Univariable and multivariable linear regressions were performed to identify which variables (“sex”, “age”, “employment”, “loneliness in the role”, “hours per day caring”, “no support for caring”, “cohabiting”, “physical exercise”, “healthy eating”, “leisure/relaxation activity”, “social activity”, “positive mental health”, “psychological vulnerability”) explained the informal caregivers’ depression, anxiety, stress symptoms, and burden.

Initially, univariate linear regressions were carried out, considering the dependent variable separately (“depression”, “anxiety”, “stress”, and “burden”) and each of the other variables (“sex”, “age”, “employment”, “loneliness in the role”, “hours per-day caring”, “no support for caring”, “cohabiting”, “physical exercise”, “healthy eating”, “leisure/relaxation activity”, “social activity”, “positive mental health”, “psychological vulnerability”) as covariates. All the covariates that showed a statistically significant association with the outcome (*p* < .05) were subsequently selected for inclusion in the multivariate model. The multivariate model was then run.

## Results

### Participant and descriptive data

The participants in this study were 414 informal caregivers of people with dementia living at home (Table 1). They were mostly daughters (58.7%), had approximately 10 ± 5.1 years of schooling, on average 60 ± 12.2 years old, married (71.7%), more than half living with the care recipient (68.1%). They had assumed caregiving role about 5 ± 4.1 years earlier and more than half (60%) were the sole informal caregivers, spending nearly to 10 ± 8.2 h per day on caregiving tasks. When they had doubts about caregiving, they turned to health professionals for clarification (*N* = 370; 89.4%) and were mainly followed by neurologists (*N* = 278; 67.1%) and mental health nurses (*N* = 132; 31.9%). Some consultations took place at home (*N* = 183; 44.2%).

**Table 1:** Descriptive characteristics of informal caregivers (*n* = 414)

	Range	Mean (SD)	*N* (%)	
**Age**	22–91	59.8 (12.2)		
**Sex**: female			336	(81.2)
**Marital status**:				
Married			297	(71.7)
Divorced			55	(13.3)
Single			47	(11.4)
Widowed			15	(3.6)
**Education years at school**	1–20	10.35 (5.1)		
**employment**:				
Employee			164	(39.6)
Retired			141	(34.1)
Stop working to care			48	(11.6)
Self-employed			26	(6.3)
Housekeeper			15	(3.6)
Unemployed			13	(3.1)
Stop work due to disease			7	(1.7)
**Relationship**:				
Child			243	(58.7)
Wife			116	(28.0)
Son / daughter-in-law			25	(6.0)
**Years as a caregiver**	0–30	4.98 (4.1)		
**Loneliness in the role**			249	(60.1)
**Hours per day caring**	0–24	10 (8.2)		
**Distance from home**:				
Cohabiting			282	(68.1)
Live in the same locality			63	(15.2)
**Care accompanied by HP**:				
Neurologist			278	(67.1)
MH nurse			132	(31.9)
Psychiatrist			64	(15.5)
**Sleep satisfaction**:				
Strongly disagree			147	(35.5)
Partially disagree			62	(15.0)
Neither agree nor disagree			35	(8.5)
Partially agree			93	(22.5)
Strongly agree			77	(18.6)
**Physical activity**:				
Daily			55	(13.3)
2/3 days week			85	(20.5)
Weekly			52	(12.6)
None			222	(53.6)
**Healthy eating**:				
Strongly agree			190	(45.9)
Partially agree			121	(29.2)
Neither agree nor disagree			36	(8.7)
Partially disagree			42	(10.1)
Strongly disagree			25	(6.0)
**Leisure/relaxation activity**:				
Daily			132	(31.9)
2/3 days week			38	(9.2)
Weekly			74	(17.9)
Monthly			25	(6.0)
None			145	(35.5)
**Social activity**:				
+ 3 days week			35	(8.5)
2/3 days week			43	(10.4)
Weekly			84	(20.3)
1/2 days monthly			109	(26.3)
None			143	(34.5)
**No diagnosis of mental illness**			338	(81.6)
Diagnosis of depression			47	(11.4)
**Positive Mental Health** (PMHQ)	82–156	129.1 (15.7)		
High level			318	(76.8%)
Moderate level			96	(23.2%)
**Psychological vulnerability** (PVS)	6–30	14.8 (5.2)		
**Depression Symptoms** (DASS21-D)	0–21	3.5 (4.6)		
**Anxiety Symptoms** (DASS21-A)	0–21	3.9 (4.6)		
**Stress Symptoms** (DASS21-S)	0–21	5.8 (5.1)		
**Caregiver’s Burden** (CBS)	22–107	54.3 (17.8)		

Note: HP – health professional. MH – mental health. SD – standard deviation. PMHQ – Positive Mental Health Questionnaire. PVS – Psychological Vulnerability Scale. DASS21 – Depression Anxiety Stress Scale-21 items. D – Depression. A – Anxiety. S – Stress. CBS – Caregiver Burden Scale

### Caregivers’ mental health

The majority of the participants (*N* = 338; 81.6%) reported having no diagnosed mental illness. However, 47 caregivers (11%) indicated that they had received a clinical diagnosis of depression from a psychiatrist. Furthermore, 137 participants (33.1%) had previously received psychological or psychiatric treatment, and 64 caregivers (15.5%) had visited the emergency department due to a mental health problem in the preceding three months. Additionally, 86 caregivers (20.8%) reported having missed their own medical treatments or appointments because no one was available to look after the person they cared for.

According to the mean score obtained on the PVS (14.8 ± 5.2; range 6–30) the participants demonstrated a moderate level of susceptibility to negative emotional experiences and psychological difficulties.

The PMHQ results showed the mean score of 129.1 ± 15.7 (range 82–156) among the informal caregivers, with 318 (76.8%) participants demonstrating a high level of positive mental health and 96 (23.2%) showing a moderate level of positive mental health.

Based on DASS-21, more them half of participants self-reported symptoms of depression (*N* = 297; 71.7%), anxiety (*N* = 259; 62.6%), and stress (*N* = 297; 71.7%) within the normal range.

Regarding the CBS results with the mean 54.3 ± 17.8 (range 22–107), 147 (35.5%) of participants experienced no burden, 90 (21.7%) mild burden, and 177 (42.8%) reported a severe burden.

### Associated factors of caregivers’ mental health

Four univariate linear regressions and statistically significant relationships were obtained (*p* < .05). Significant relationships were found with the variables: “sex”, “loneliness in the role”, “hours per day caring”, “physical activity”, “healthy eating”, “leisure/relaxation activity”, “social activity”, “psychological vulnerability”, and “positive mental health”, assuming “depression” as the dependent variable. For the outcome variable “anxiety”, significant relationships were established with “sex”, “employed”, “loneliness in the role”, “hours per day caring”, “healthy eating”, “leisure/relaxation activity”, “social activity”, “psychological vulnerability”, and “positive mental health”. For the dependent variable “stress”, significant relationships were observed with “sex”, “healthy eating”, “leisure/relaxation activity”, “social activity”, “psychological vulnerability”, and “positive mental health”. The final univariate linear regression established a significant relationship between the outcome variable “burden” and the variables: “sex”, “age”, “loneliness in the role”, “years as a caregiver”, “hours per day caring”, “physical activity”, “healthy eating”, “leisure/relaxation activity”, “social activity”, “psychological vulnerability”, and “positive mental health”.

Prior to conducting the regression analyses, we examined the correlation between anxiety and depression using Pearson’s correlation coefficient (two-tailed). The analysis revealed a strong positive correlation between anxiety and depression (*r* = .799, *p* < .001), indicating that higher levels of anxiety were closely associated with higher levels of depression among caregivers.

Tables 2, 3, 4 and 5 show the four multivariate linear regression models that include only the previously statistically significant variables associated with the four dependent variables under study (“depression”, “anxiety”, “stress”, and “burden”). Table 2, presented below, shows the multivariate linear regression carried out with “Depression” symptoms as the outcome variable. This model was statistically significant, *F*(8, 405) = 20.50, *p* < .001, and accounted for 27.9% of the variance (adjusted R² = 0.279). Increased daily caregiving hours were positively associated with higher depressive symptoms scores (*B* = 0.050, *p* = .037). In contrast, engaging in leisure or relaxation activities on a daily basis was significantly associated with lower depressive symptoms (*B* = − 1.403, *p* = .006). Weekly social activity was also protective, with caregivers participating two to three times per week reporting fewer depressive symptoms (*B* = − 1.457, *p* = .042). Moreover, higher psychological vulnerability was positively related to depressive symptoms (*B* = 0.289, *p* < .001), while greater positive mental health was inversely associated (*B* = –0.061, *p* < .001).

**Table 2:** Multivariate linear regression

DASS21-D^a^	B	95% CI	*p*
**Hours per day caring**	0.050	[0.003; 0.097]	0.037
**Leisure/ relaxation activity**:			
Daily	-1.403	[-2.400; − 0.405]	0.006
2/3 days week	− 0.537	[-1.998; 0.924]	0.470
Weekly	− 0.426	[-1.551; 0.700]	0.457
Monthly	0.690	[-0.986; 2.366]	0.419
**Social activity**			
+3 days a week	− 0.934	[-2.450; 0.581]	0.226
2 to 3 days a week	-1.457	[-2.860; − 0.055]	0.042
Weekly	− 0.957	[-2.055; 0.140]	0.087
Monthly	− 0.685	[-1.698; 0.328]	0.185
**PMHQ**	− 0.061	[-0.087; − 0.036]	< 0.001
**PVQ**	0.289	[0.211; 0.368]	< 0.001

Note: a) Adjusted R-squared = 0.279. CI = confidence interval for B. ANOVAa *p* < .001

PMHQ – Positive Mental Health Questionnaire. PVQ – Psychological Vulnerability Scale. DASS21 – Depression Anxiety Stress Scale-21 items. D – Depressions

Outcome variable: depression symptoms (*N* = 414)

The results of multivariate linear regression carried out with “anxiety” symptoms as the outcome variable are presented in Table 3. This model was significant, *F*(9, 404) = 14.47, *p* < .001, explaining 23.2% of the variance (adjusted R² = 0.232). Female caregivers reported significantly higher anxiety than males (*B* = − 1.145, *p* = .029), whereas being employed was associated with reduced anxiety (*B* = − 1.034, *p* = .016). Caregivers who strongly adhered to healthy eating habits reported lower anxiety (*B* = − 2.056, *p* = .019). Likewise, frequent social activity, particularly two to three times per week, was negatively associated with anxiety (*B* = − 1.878, *p* = .009). Psychological vulnerability was positively associated with higher anxiety levels (*B* = 0.272, *p* < .001), whereas positive mental health was inversely associated (*B* = –0.036, *p* = .011).

**Table 3:** Multivariate linear regression

DASS21-A^a^	B	95% CI	*p*
**Sex**	-1.145	[-2.170; − 0.119]	0.029
**Employment**	-1.034	[-1.872; − 0.197]	0.016
**Healthy eating**:			
Strongly agree	-2.056	[-3.778; − 0.335]	0.019
Partially agree	− 0.770	[-2.548; 1.008]	0.395
Neither agree nor disagree	− 0.221	[-2.301; 1.859]	0.834
Partially disagree	− 0.754	[-2.784; 1.276]	0.466
**Social activity:**			
+3 days week	0.1.396	[-2.916; 0.123]	0.071
2 to 3 days a week	-1.878	[-3.284; − 0.472]	0.009
Weekly	-1.399	[-2.526; − 0.272]	0.015
1 to 2 monthly	− 0.627	[-1.691; 0.437]	0.247
**PMHQ**	− 0.036	[-0.064; − 0.008]	0.011
**PVQ**	0.272	[0.190; 0.354]	< 0.001

Note: a) Adjusted R-squared = 0.232. CI = Confidence Interval for B. ANOVA^a^ *p* < .001

PMHQ – Positive Mental Health Questionnaire. PVQ – Psychological Vulnerability Scale. DASS21 – Depression Anxiety Stress Scale-21 items. A – Anxiety

Outcome variable: anxiety symptoms (*N* = 414)

Table 4 shows the multivariate linear regression carried out with “stress” symptoms as the outcome variable. This regression model was significant, *F*(8, 405) = 17.47, *p* < .001, accounting for 24.2% of the variance (adjusted R² = 0.242). Female caregivers reported higher stress (*B* = − 1.519, *p* = .007). Strongly agree with the practice of healthy eating habits was associated with lower stress (*B* = − 2.060, *p* = .031). Regular social activity (two to three times per week: *B* = − 1.997, *p* = .011; weekly: *B* = − 1.596, *p* = .010) was also associated with lower stress levels. As with other models, psychological vulnerability was positively associated with stress (*B* = 0.347, *p* < .001), while positive mental health was inversely associated (*B* = –0.035, *p* = .024).

**Table 4:** Multivariate linear regression

DASS21-S^a^	B	95% CI	*p*
**Sex**	-1.519	[-2.630; − 0.409]	0.007
**Healthy eating**:			
Strongly agree	-2.060	[-3.937; − 0.184]	0.031
Partially agree	-1.088	[-3.024; 0.849]	0.270
Neither agree nor disagree	− 0.467	[-2.733; 1.798]	0.685
Partially disagree	-1.143	[-3.355; 1.070]	0.311
**Social activity:**			
+3 days week	-1.453	[-3.106; 0.199]	0.085
2 to 3 days week	-1.997	[-3.528; − 0.466]	0.011
Weekly	-1.596	[-2.806; − 0.386]	0.010
1 to 2 monthly	− 0.517	[-1.639; 0.605]	0.366
**PMHQ**	− 0.035	[-0.065; − 0.005]	0.024
**PVQ**	0.347	[0.258; 0.436]	< 0.001

Note: a) Adjusted R-squared = 0.242. CI = Confidence Interval for B. ANOVA^a^ *p* < .001

PMHQ – Positive Mental Health Questionnaire. PVQ – Psychological Vulnerability Scale; DASS21 – Depression Anxiety Stress Scale-21 items. S – Stress

Outcome variable: stress symptoms (*N* = 414)

Table 5 shows the multivariate linear regression carried out with “caregiver burden” as the outcome variable. This model was statistically significant, *F*(9, 404) = 18.44, *p* < .001, explaining 27.3% of the variance (adjusted R² = 0.273). Younger age was associated with higher burden (*B* = –0.191, *p* = .007), as were more years in the caregiving role (*B* = 0.505, *p* = .007) and longer daily caregiving hours (*B* = 0.270, *p* = .009). Caregivers who reported loneliness in their role also had significantly higher burden (*B* = 3.268, *p* = .050). Conversely, engaging daily in leisure or relaxation activities was strongly protective (*B* = − 10.031, *p* < .001). Consistent with other outcomes, psychological vulnerability was positively associated with burden (*B* = 0.870, *p* < .001), while positive mental health was inversely related (*B* = –0.175, *p* < .001).

**Table 5:** Multivariate linear regression

CBS^a^	B	95% CI	*p*
**Age**	− 0.191	[-0.329; − 0.052]	0.007
**Loneliness in the role**	3.268	[0.006; 6.530]	0.050
**Years as a caregiver**	0.505	[0.136; 0.875]	0.007
**Hours per day caring**	0.270	[0.068; 0.471]	0.009
**Leisure/ relaxation activity**:			
Daily	-10.031	[-13.795; -6.267]	< 0.001
2/3 days week	-2.235	[-7.717; 3.247]	0.423
Weekly	-2.115	[-6.447; 2.217]	0.338
Monthly	− 0.835	[-7.344; 5.674]	0.801
**PMHQ**	− 0.175	[-0.276; − 0.074]	< 0.001
**PVQ**	0.870	[0.563; 1.177]	< 0.001

Note: a) Adjusted R-squared = 0.273. CI = Confidence Interval for B. ANOVA^a^ *p* < .001

CBS – Caregiver Burden Scale. PMHQ – Positive Mental Health Questionnaire. PVQ – Psychological Vulnerability Scale

Outcome variable: burden (*N* = 414)

## Discussion

This study aimed to identify levels of anxiety, stress, depressive symptoms, and caregiver burden, as well as the sociodemographic and clinical factors influencing these outcomes among Portuguese informal caregivers of people with dementia. The sample reflected patterns previously reported in Portugal most caregivers were daughters, middle-aged, married, and with relatively high education. Over two-thirds cohabited with the care recipient, and 45.9% were employed or self-employed and used formal services [49, 58, 59]. By contrast, Gonçalves-Pereira et al. (2019) found a predominance of wives as caregivers, lower education, and less use of formal services. Since 2019, Portugal has formally recognised and regulated the status of informal caregivers, strengthening their social and economic rights, including access to respite support. Male participation in caregiving is gradually increasing [7, 59, 70]. This study reflects a Southern European pattern characterised by strong familism (family loyalty and solidarity), co-residence, and long hours of caregiving [49, 59, 71, 72]. A previous study has shown that informal caregivers often face difficulties in balancing care for ageing parents with the responsibilities of raising their own children [49].

Across Europe, dementia-friendly policies, health expenditure, and service coverage are generally higher in Northern, Western, and Central countries than in Eastern regions, where support remains underdeveloped [57–77]. In Sweden, broader formal service provision is associated with fewer caregiving hours and lower caregiver burden compared with Mediterranean contexts [54].

In the African continent, studies from Ghana indicate that most caregivers are daughters who frequently co-reside with the care recipient, with caregiving shaped by religion and ethnicity [78]. In Arab Muslim societies, religious and spiritual values reinforce traditions of honouring and caring for parents, while in Egypt, where extended families are common, caregiving is regarded as a collective responsibility shared among family members [79]. Compared with caregivers in Ghana, the Portuguese were generally older and had higher educational attainment. The majority of Egyptian caregivers belong to extended families, which provide mutual support in caregiving, while the Portuguese assumed the caregiving role alone (60.1%).

In Asian contexts such as China, Taiwan, South Korea, and Japan, caregiving is predominantly assumed by family members, with greater male involvement than in Europe. It is framed as both a responsibility and a moral obligation, reinforced by filial piety and even stigma [80–85]. These cultural values are linked to lower use of formal services, delayed institutionalisation, and perceptions of caregiving as less psychologically threatening, as it preserves the patient’s identity and provides intrinsic rewards [80]. Caregivers in Asian countries also report lower burden than in American and European settings, reflecting cultural views of caregiving as a natural life role rather than a disruption [83].

Leggins et al. (2024), in an American study of caregivers, identified ethnic differences in caregiving. Hispanic caregivers were generally younger, Asian caregivers were predominantly men and almost exclusively family members, whereas White, Black, and multiracial caregivers more often included non-family members, such as friends or neighbours [86]. Cultural values can be understood through three dimensions - familism, religious or philosophical beliefs, and filial piety - which, shaped by cultural norms, reinforce caregivers’ sense of duty and moral obligation to provide care [60].

Most participants presented a high level of positive mental health, similar to findings in a randomized controlled trial study conducted with Spanish caregivers, which confirm the protective role of positive mental health [87]. A scoping review by Andrade and colleagues (2022) highlighted a significant research gap in the positive mental health of family caregivers [32]. The PMHQ featured questions that can reflect the positive outcomes of caregiving, including satisfaction, mastery and fulfillment, self-efficacy, meaning in life, personal growth, improved social connections, recognition of personal strength, problem-solving perspectives, and valuing positive experiences [88, 89].

Regarding mental health, only a minority of caregivers presented clinically relevant symptoms of depression, anxiety, and stress, consistent with findings in other Portuguese samples [7]. In this study sex, leisure or relaxation activities, social interactions outside of the caregiving context, psychological vulnerability, and levels of positive mental health are significant factors, whereas Kurten (2021), identified time spent supervising the patient with dementia among German caregivers, predominantly spouses and more often urban, of a higher risk of depression. The urban–rural dimension was not assessed in the present study [20].

Caregivers who exhibit resilience tend to experience lower levels of depression, improved overall health, positive social support, better self-esteem, and greater satisfaction and achievement [89]. Family members and friends play a crucial role in facilitating the expression of concerns and emotions and collaboratively fostering problem-solving [90]. Leisure activity programs for caregivers, demonstrating their effectiveness in reducing depressive symptoms. These programs allow caregivers to focus on activities unrelated to caregiving, alleviating some of the burden [40].

Sex, social interactions outside the caregiving context, psychological vulnerability, and levels of positive mental health were associated with anxiety and stress symptoms among the participants. Compared with their male counterparts, female caregivers tend to spend more time providing care and experience greater tension and role conflict [46, 91]. Additionally, significant others, including friends, neighbours, and work colleagues, can provide crucial emotional support and encouragement [15, 90]. Mentally vulnerable individuals reported more symptoms and diseases, utilised health and social services, and had less healthy lifestyles [92].

In this study, 35.5% of the caregivers reported no burden, 21.7% mild burden, and 42.8% severe burden. Similar results were presented in other study [59]; however, higher burden values were obtained in another Portuguese study [49]. Age, years as a caregiver, daily care hours, time allocated for leisure/relaxation, psychological vulnerability, and positive mental health were identified as significant factors to caregiver burden. These findings are consistent with previous research observations [23, 46, 50, 93–96].

### Limitations

Several limitations regarding the results must be acknowledged. First, the study’s cross-sectional design restricts our ability to infer causality. Second, the non-randomised sampling approach limits the generalisability of the findings to the broader population of Portuguese caregivers of dementia patients. Third, the study does not account for the potential confounding effects of professional guidance and counselling that some caregivers may have received. Fourth, the exclusive focus on caregivers and the requirement of at least one hour of weekly contact with the care recipient, although both methodologically justified, may have underestimated caregiver burden.

### Practical implications

The training of nursing professionals should include education on the Multifactorial Model of Positive Mental Health, the development of skills to assess positive mental health, and preparation to implement promotion programmes for informal caregivers of people with dementia. Because cultural perspective shapes the caregiving experience, nursing formation should also integrate transcultural competencies, equipping future professionals to deliver culturally appropriate interventions tailored to the caregiver’s context. In clinical practice, the systematic assessment of caregivers’ positive mental health and psychological vulnerability should be incorporated into routine care, enabling early identification of needs and timely informal caregivers’ support. Nurses are well positioned to implement structured interventions, such as psychoeducational sessions based on this model, leisure and relaxation programmes, and peer-support groups, which can strengthen resilience and mitigate caregiver burden.

## Conclusion

This study provides evidence on the mental health of Portuguese informal caregivers of people with dementia, highlighting both risk and protective factors. While a considerable proportion of caregivers reported no burden and a slightly higher proportion reported severe burden, most did not present clinically relevant symptoms of depression, anxiety, or stress, confirming the heterogeneity of caregiving experiences at both national and international levels. Greater time dedicated to caregiving and reduced engagement in leisure or relaxation activities were significantly associated with higher burden, whereas positive mental health emerged as a key protective factor. These findings emphasise the importance of health professionals who work with informal caregivers regularly assessing their positive mental health and psychological vulnerability. Interventions centred on promoting positive mental health may help mitigate emotional symptoms and caregiver burden, representing a promising avenue of support. Furthermore, a more detailed exploration of the predictive capacity of PMHQ subscales would be valuable in future research, particularly regarding resilience dimensions in Portugal. Replication of this study in different cultural and geographical contexts, where demographic ageing is expected to increase dementia prevalence, may also be recommended.

## Data Availability

The datasets used and/or analysed in this study are available from the corresponding author upon reasonable request.

## References

[1] Eurostat. Ageing europe: looking at the lives of older people in the EU. Luxembourg: Publications Office of the European Union; 2020. 10.2785/628105.

[2] OECD. Health at a glance: OECD indicators. Paris: OECD Publishing; 2021. 10.1787/4dd50c09-en.

[3] Börsch-Supan A, Douhou S, Otero MC, Tawiah BB. Harmonized prevalence estimates of dementia in Europe vary strongly with childhood education. Sci Rep. 2025. 10.1038/s41598-025-97691-z. 40269049 10.1038/s41598-025-97691-z PMC12019132

[4] Alzheimer Europe. Dementia in Europe Yearbook 2019: Estimating the prevalence of dementia in Europe. Luxembourg: Alzheimer Europe. 2019. ISBN 978-99959-995-9-9.

[5] Gonçalves-Pereira M, Verdelho A, Prina M, Marques MJ, Xavier M. How many people live with dementia in Portugal? A discussion paper of national estimates. Port J Public Health. 2021;39(1):58–68. 10.1159/000516503 10.1159/000516503 PMC11320114 39469039

[6] Angeles RC, Berge LI, Gedde MH, Kjerstad E, Vislapuu M, Puaschitz NG, et al. Which factors increase informal care hours and societal costs among caregivers of people with dementia? A systematic review of resource utilization in dementia (RUD). Health Econ Rev. 2021;11(1):37. 10.1186/s13561-021-00333-z. 34536149 10.1186/s13561-021-00333-z PMC8449888

[7] Gonçalves-Pereira M, Marques MJ, Balsinha C, Fernandes A, Machado AS, Verdelho A, et al. Necessidades de Cuidados e Recurso aos Serviços na Demência: Avaliação Inicial da Coorte Portuguesa no Estudo Actifcare. Acta Med Port [Internet]. 2019;32(5):355. Available from: https://www.actamedicaportuguesa.com/revista/index.php/amp/article/view/11136 10.20344/amp.11136 31166896

[8] Kraijo H, van Exel J, Brouwer W. The perseverance time of informal carers for people with dementia: results of a two-year longitudinal follow-up study. BMC Nurs. 2015;14(1):10. 10.1186/s12912-015-0107-5. 26549986 10.1186/s12912-015-0107-5 PMC4636746

[9] United Nations Economic Commission for Europe. The challenging roles of informal carers. Policy brief on Ageing, No. 22. Geneva: UNECE; 2019.

[10] Alzheimer’s Disease International. World alzheimer report 2024: global changes in attitudes to dementia. London: Alzheimer’s Disease International; 2024.

[11] Bauer JM, Sousa-Poza A. Impacts of informal caregiving on caregiver employment, health, and family. J Popul Ageing. 2015;8(3):113–45. 10.1007/s12062-015-9116-0.

[12] Webster C, Morais J. Interventions for the carers. In: Gauthier S, Webster C, Servaes S, Morais JA, Rosa-Neto P, editors. World alzheimer report 2022: life after diagnosis: navigating treatment, care and support. London: Alzheimer’s Disease International; 2022. pp. 261–82.

[13] Yu DSF, Cheng ST, Wang J. Unravelling positive aspects of caregiving in dementia: an integrative review of research literature. Int J Nurs Stud. 2018;79:1–26. 10.1016/j.ijnurstu.2017.10.008. 29128685 10.1016/j.ijnurstu.2017.10.008

[14] Aranda MP, Kremer IN, Hinton L, Zissimopoulos J, Whitmer RA, Hummel CH, et al. Impact of dementia: health disparities, population trends, care interventions, and economic costs. J Am Geriatr Soc. 2021;69(7):1774–83. 10.1111/jgs.17345. 34245588 10.1111/jgs.17345 PMC8608182

[15] Del-Pino-Casado R, Cardosa MR, López-Martínez C, Orgeta V. The association between subjective caregiver burden and depressive symptoms in carers of older relatives: a systematic review and meta-analysis. PLoS ONE. 2019;14(5):e0217648. 10.1371/journal.pone.0217648. 31141556 10.1371/journal.pone.0217648 PMC6541277

[16] Ervin J, Taouk Y, Fleitas Alfonzo L, Peasgood T, King T. Longitudinal association between informal unpaid caregiving and mental health amongst working-age adults in high-income OECD countries: a systematic review. EClinicalMedicine. 2022;53:101711. 10.1016/j.eclinm.2022.101711. 36353526 10.1016/j.eclinm.2022.101711 PMC9637877

[17] Gilsenan J, Gorman C, Shevlin M. Explaining caregiver burden in a large sample of UK dementia caregivers: the role of contextual factors, behavioural problems, psychological resilience, and anticipatory grief. Aging Ment Health. 2023;27(7):1274–81. 10.1080/13607863.2022.2102138. 35881027 10.1080/13607863.2022.2102138

[18] Karg N, Graessel E, Randzio O, et al. Dementia as a predictor of care-related quality of life in informal caregivers: a cross-sectional study to investigate differences in health-related outcomes between dementia and non-dementia caregivers. BMC Geriatr. 2018;18:189. 10.1186/s12877-018-0885-1. 30139354 10.1186/s12877-018-0885-1 PMC6108112

[19] Kim Y, Schulz R. Family caregivers’ strains: comparative analysis of cancer caregiving with dementia, diabetes, and frail elderly caregiving. J Aging Health. 2008;20(5):483–503. 10.1177/0898264308317533. 18420838 10.1177/0898264308317533

[20] Kürten L, Dietzel N, Kolominsky-Rabas PL, Graessel E. Predictors of the one-year-change in depressiveness in informal caregivers of community-dwelling people with dementia. BMC Psychiatry. 2021;21:177. 10.1186/s12888-021-03164-8. 33812389 10.1186/s12888-021-03164-8 PMC8019174

[21] Papastavrou E, Charalambous A, Tsangari H, Karayiannis G. The burdensome and depressive experience of caring: what cancer, schizophrenia, and alzheimer’s disease caregivers have in common. Cancer Nurs. 2012;35(3):187–94. 10.1097/NCC.0b013e31822cb4a0. 22538260 10.1097/NCC.0b013e31822cb4a0

[22] Parker LJ, Fabius C, Rivers E, Taylor JL. Is dementia-specific caregiving compared with non-dementia caregiving associated with physical difficulty among caregivers for community-dwelling adults? J Appl Gerontol. 2022;41(4):1074–80. 10.1177/07334648211014352. 34041929 10.1177/07334648211014352 PMC8664093

[23] Steinsheim G, Malmedal W, Follestad T, Olsen B, Saga S. Factors associated with subjective burden among informal caregivers of home-dwelling people with dementia: a cross-sectional study. BMC Geriatr. 2023;23(1):358. 10.1186/s12877-023-04358-3. 37817101 10.1186/s12877-023-04358-3 PMC10565959

[24] Wawrziczny E, Berna G, Ducharme F, Kergoat MJ, Pasquier F, Antoine P. Characteristics of the spouse caregiving experience: comparison between early- and late-onset dementia. Aging Ment Health. 2018;22(9):1207–15. 10.1080/13607863.2017.1339777. 28631510 10.1080/13607863.2017.1339777

[25] George LK, Gwyther LP. Caregiver well-being: a multidimensional examination of family caregivers of demented adults. Gerontologist. 1986;26:p253. 10.1093/geront/26.3.253 3721232

[26] Pearlin LI, Mullan JT, Semple SJ, Skaff MM. Caregiving and the stress process: an overview of concepts and their measures. Gerontologist. 1990;30(5):583–94. 10.1093/geront/30.5.583. 2276631 10.1093/geront/30.5.583

[27] Su YY, Wang WF, Jhang KM, et al. Enhancing care service utilization and reducing burden: the role of needs assessments for dementia caregivers in long-term care. BMC Nurs. 2025;24:1004. 10.1186/s12912-025-03686-6. 40751254 10.1186/s12912-025-03686-6 PMC12317630

[28] World Health Organization. The world health report 2001: mental health – new understanding, new hope. Geneva: World Health Organization; 2001. https://iris.who.int/handle/10665/42390.

[29] Jahoda M. Current concepts of positive mental health. New York: Basic Books; 1958.

[30] Lluch MT. Concepto de Salud mental positiva: factores relacionados. In: Fornés J, Gómez J, editors. Recursos y programas Para La Salud mental. Enfermería psicosocial II. Madrid: Fuden; 2008. pp. 37–68.

[31] Sequeira C, Carvalho JC, Roldan-Merino N, Moreno-Poyato AR, Teixeira S, David B, Costa PS, Puig-Llobet M, Lluch-Canut MT. Psychometric properties of the positive mental health questionnaire: short form (PMHQ-SF18) in young adults. Front Public Health. 2024;12:1375378. 10.3389/fpubh.2024.1375378. 38799675 10.3389/fpubh.2024.1375378 PMC11122894

[32] Andrade C, Tavares M, Soares H, Coelho F, Tomás C. Positive mental health and mental health literacy of informal caregivers: a scoping review. Int J Environ Res Public Health. 2022;19(22):15276. 10.3390/ijerph192215276. 36430000 10.3390/ijerph192215276 PMC9690944

[33] Ferré-Bergadà M, Valls A, Raigal-Aran L, Lorca-Cabrera J, Albacar-Riobóo N, Lluch-Canut T, et al. A method to determine a personalized set of online exercises for improving the positive mental health of a caregiver of a chronically ill patient. BMC Med Inf Decis Mak. 2021;21(1):45. 10.1186/s12911-021-01445-6. 10.1186/s12911-021-01445-6 PMC7905974 33632207

[34] Ferré-Grau C, Raigal-Aran L, Lorca-Cabrera J, Ferré-Bergadà M, Lleixà-Fortuño M, Lluch-Canut MT, et al. A multi-centre, randomized, 3-month study to evaluate the efficacy of a smartphone app to increase caregivers’ positive mental health. BMC Public Health. 2019;19(1):888. 10.1186/s12889-019-7264-5. 31277623 10.1186/s12889-019-7264-5 PMC6612114

[35] Sequeira CA, Barbosa EN, Nogueira MJ, Sampaio FM. Evaluation of the psychometric properties of the mental vulnerability questionnaire in undergraduate students. Perspect Psychiatr Care. 2017;53(4):243–50. 10.1111/ppc.12164. 27198975 10.1111/ppc.12164

[36] Teixeira S, Ferré-Grau C, Canut TL, Pires R, Carvalho JC, Ribeiro I, et al. Positive mental health in university students and its relations with psychological vulnerability, mental health literacy, and sociodemographic characteristics: a descriptive correlational study. Int J Environ Res Public Health. 2022;19(6):3185. 10.3390/ijerph19063185. 35328870 10.3390/ijerph19063185 PMC8949352

[37] Sinclair VG, Wallston KA. The development and validation of the psychological vulnerability scale. Cogn Ther Res. 1999;23(2):119–29. 10.1023/A:1018770926615.

[38] Frias CE, Risco E, Zabalegui A. Psychoeducational intervention on burden and emotional well-being addressed to informal caregivers of people with dementia. Psychogeriatrics. 2020;20(6):900–9. 10.1111/psyg.12616. 33015927 10.1111/psyg.12616

[39] Rico-Blázquez M, Escortell-Mayor E, del-Cura-González I, Sanz-Cuesta T, Gallego-Berciano P, de las Casas-Cámara G, et al. CuidaCare: effectiveness of a nursing intervention on caregivers’ quality of life: cluster-randomized clinical trial. BMC Nurs. 2014;13(1):2. 10.1186/1472-6955-13-2. 24467767 10.1186/1472-6955-13-2 PMC3915556

[40] Wiegelmann H, Speller S, Verhaert LM, Schirra-Weirich L, Wolf-Ostermann K. Psychosocial interventions to support the mental health of informal caregivers of persons living with dementia – a systematic literature review. BMC Geriatr. 2021;21(1):20. 10.1186/s12877-021-02020-4. 33526012 10.1186/s12877-021-02020-4 PMC7849618

[41] Zegwaard MI, Aartsen MJ, Grypdonck MH, Abma TA. Trust: an essential condition in the application of a caregiver support intervention in nursing practice. BMC Psychiatry. 2017;17(1):47. 10.1186/s12888-017-1209-2. 28148235 10.1186/s12888-017-1209-2 PMC5288942

[42] Chen YJ, Su JA, Chen JS, Liu CH, Griffiths MD, Tsai HC, et al. Examining the association between neuropsychiatric symptoms among people with dementia and caregiver mental health: are caregiver burden and affiliate stigma mediators? BMC Geriatr. 2023;23(1):42. 10.1186/s12877-023-03735-2. 36646996 10.1186/s12877-023-03735-2 PMC9841634

[43] Sołtys A, Tyburski E. Predictors of mental health problems in formal and informal caregivers of patients with alzheimer’s disease. BMC Psychiatry. 2020;20(1):435. 10.1186/s12888-020-02822-7. 32887576 10.1186/s12888-020-02822-7 PMC7487573

[44] Boucher A, Haesebaert J, Freitas A, Adekpedjou R, Landry M, Bourassa H, et al. Time to move? Factors associated with burden of care among informal caregivers of cognitively impaired older people facing housing decisions: secondary analysis of a cluster randomized trial. BMC Geriatr. 2019;19(1):249. 10.1186/s12877-019-1249-1. 31500590 10.1186/s12877-019-1249-1 PMC6734334

[45] de Graaff F, Joling KJ, van Broese MI, Gerritsen DL, de Lange J, de Valk MM, et al. Is caregiver burden associated with sex and gender-related characteristics? A large-scale survey study among family caregivers of people with dementia. BMC Geriatr. 2025;25:171. 10.1186/s12877-025-05795-y. 40087581 10.1186/s12877-025-05795-y PMC11908074

[46] García-Martín V, de Hoyos-Alonso MC, Delgado-Puebla R, Ariza-Cardiel G, del Cura-González I. Burden in caregivers of primary care patients with dementia: influence of neuropsychiatric symptoms according to disease stage (NeDEM project). BMC Geriatr. 2023;23:525. 10.1186/s12877-023-04234-0. 37644410 10.1186/s12877-023-04234-0 PMC10463529

[47] Jawahir S, Tan EH, Tan YR, Mohd Noh SN, Ab Rahim I. The impacts of caregiving intensity on informal caregivers in malaysia: findings from a National survey. BMC Health Serv Res. 2021;21:391. 10.1186/s12913-021-06412-5. 33906646 10.1186/s12913-021-06412-5 PMC8077883

[48] Sabatini S, Clare L, Victor C, Matthews F, Martyr A. Health conditions in spousal caregivers of people with dementia and their relationships with stress, caregiving experiences, and social networks: longitudinal findings from the IDEAL programme. BMC Geriatr. 2024;24:171. 10.1186/s12877-024-04707-w. 38373905 10.1186/s12877-024-04707-w PMC10875834

[49] Teles S, Viana J, Freitas A, et al. Predicting informal dementia caregivers’ desire to institutionalize through mining data from an eHealth platform. BMC Geriatr. 2024;24:721. 10.1186/s12877-024-05128-5. 39210277 10.1186/s12877-024-05128-5 PMC11363529

[50] Kim H, Chang M, Rose K, Kim S. Predictors of caregiver burden in caregivers of individuals with dementia. J Adv Nurs. 2012;68(4):846–55. 10.1111/j.1365-2648.2011.05787.x. 21793872 10.1111/j.1365-2648.2011.05787.x

[51] Leocadie MC, Morvillers JM, Pautex S, Rothan-Tondeur M. Characteristics of the skills of caregivers of people with dementia: observational study. BMC Prim Care. 2020;21(1):149. 10.1186/s12875-020-01218-6. 10.1186/s12875-020-01218-6 PMC7382844 32711454

[52] Song MJ, Kim JH. Family caregivers of people with dementia have poor sleep quality: a nationwide population-based study. Int J Environ Res Public Health. 2021;18(24):13079. 10.3390/ijerph182413079. 34948685 10.3390/ijerph182413079 PMC8702002

[53] Tsapanou A, Zoi P, Sakka P. Sleep, diet, and exercise: how much dementia caregivers are affected? Brain Sci. 2024;14(8):826. 10.3390/brainsci14080826. 39199517 10.3390/brainsci14080826 PMC11352422

[54] Wulff J, Fänge AM, Lethin C, Chiatti C. Self-reported symptoms of depression and anxiety among informal caregivers of persons with dementia: a cross-sectional comparative study between Sweden and Italy. BMC Health Serv Res. 2020;20(1):15. 10.1186/s12913-020-05964-2. 33267856 10.1186/s12913-020-05964-2 PMC7709414

[55] Lindt N, van Berkel J, Mulder BC. Determinants of overburdening among informal carers: a systematic review. BMC Geriatr. 2020;20:304. 10.1186/s12877-020-01708-3. 32847493 10.1186/s12877-020-01708-3 PMC7448315

[56] Lou Q, Liu S, Huo YR, Liu M, Liu S, Ji Y. Comprehensive analysis of patient and caregiver predictors for caregiver burden, anxiety and depression in alzheimer’s disease. J Clin Nurs. 2015;24(17–18):2668–78. 10.1111/jocn.12870. 26108739 10.1111/jocn.12870

[57] Alzheimer Europe. European dementia monitor 2023: comparing and benchmarking National dementia strategies and policies. Luxembourg: Alzheimer Europe; 2023.

[58] Barbosa F, Voss G, Delerue Matos A. Health impact of providing informal care in Portugal. BMC Geriatr. 2020;20:440. 10.1186/s12877-020-01841-z. 33131486 10.1186/s12877-020-01841-z PMC7603691

[59] Lopes H, editor. Viver com Demência: Um Olhar sobre a Vida dos Cuidadores Informais de Pessoas com Demência em Portugal. Lisboa: NOVA IMS, Universidade Nova de Lisboa; NOVA Center for Global Health; 2024. ISBN: 978-972-8093-24-2.

[60] Zarzycki M, Seddon D, Bei E, Dekel R, Morrison V. How culture shapes informal caregiver motivations: a meta-ethnographic review. Qual Health Res. 2022;32(10):1574–89. 10.1177/10497323221110356. 35737473 10.1177/10497323221110356 PMC9411702

[61] von Elm E, Altman DG, Egger M, Pocock SJ, Gøtzsche PC, Vandenbroucke JP. The strengthening the reporting of observational studies in epidemiology (STROBE) statement: guidelines for reporting observational studies. Int J Surg. 2014;12(12):1495–9. 10.1016/j.ijsu.2014.07.013. 25046131 10.1016/j.ijsu.2014.07.013

[62] Sequeira C, Carvalho JC, Sampaio F, Sá L, Lluch-Canut T, Roldán-Merino J. Avaliação Das propriedades psicométricas do Questionário de Saúde mental positiva Em estudantes Portugueses do Ensino superior. Rev Port Enferm Saúde Mental. 2014;11:45–53.

[63] Sequeira C. Adaptação e validação Da Escala de sobrecarga do Cuidador de Zarit. Rev Enferm Referência. 2010;12:9–16.

[64] Pais-Ribeiro JL, Honrado A, Leal I. Contribuição Para o Estudo Da adaptação Portuguesa Das Escalas de Ansiedade, Depressão e stress (EADS) de 21 itens de lovibond e lovibond. Psicol Saúde Doenças. 2004;5(2):229–39.

[65] Lluch-Canut MT. Construction of a scale to assess positive mental health [doctoral thesis]. Barcelona: Faculty of Psychology, University of Barcelona; 1999. Available from: https://hdl.handle.net/2445/42359. ISBN: 8468863130.

[66] Lluch-Canut MT. Empirical evaluation of a conceptual model of positive mental health. Ment Health. 2002;25(4):42–55.

[67] Lovibond PF, Lovibond SH. The structure of negative emotional states: comparison of the depression anxiety stress scales (DASS) with the Beck depression and anxiety inventories. Behav Res Ther. 1995;33(3):335–43. 10.1016/0005-7967(94)00075-U. 7726811 10.1016/0005-7967(94)00075-u

[68] Zarit SH, Reever KE, Bach-Peterson J. Relatives of the impaired elderly: correlates of feelings of burden. Gerontologist. 1980;20(6):649–55. 10.1093/geront/20.6.649. 7203086 10.1093/geront/20.6.649

[69] Scazufca M, Menezes PR, Almeida OP. Caregiver burden in an elderly population with depression in São Paulo, Brazil. Soc Psychiatry Psychiatr Epidemiol. 2002;37(9):416–22. 10.1007/s00127-002-0571-6. 12242617 10.1007/s00127-002-0571-6

[70] Portugal. Ministério Das Finanças, Ministério do Trabalho, solidariedade e Segurança Social, Ministério Da Saúde. Portaria n.º 335-A/2023, de 3 de novembro. Diário Da República. 2023;1(213):12–4.

[71] Dixe MA, Querido AI. Informal caregiver of dependent person in self-care: burden-related factors. Rev Enferm Ref. 2020;5(3). 10.12707/RV20013.

[72] Teles S, Ferreira A, Seeher K, et al. Online training and support program (iSupport) for informal dementia caregivers: protocol for an intervention study in Portugal. BMC Geriatr. 2020;20:10. 10.1186/s12877-019-1364-z. 31914936 10.1186/s12877-019-1364-z PMC6950829

[73] Dibao-Dina A, Filbet M, Bagaragaza E, Njamnsu J, Rusakaniko S, Moukoko E, et al. Identifying the needs of natural caregivers caring for a person with dementia: a mixed-method study. BMC Prim Care. 2025;26:48. 10.1186/s12875-025-02724-1. 39984902 10.1186/s12875-025-02724-1 PMC11846302

[74] World Health Organization. Intersectoral global action plan on epilepsy and other neurological disorders 2022–2031. Geneva: WHO; 2023. Licence: CC BY-NC-SA 3.0 IGO.

[75] OECD Statistics. Health expenditure and financing [Internet]. 2025 [2025 Sep 2]. Available from: https://stats.oecd.org/Index.aspx?ThemeTreeId=9

[76] Kontrimiene A, Sauseriene J, Blazeviciene A, Raila G, Jaruseviciene L. Qualitative research of informal caregivers’ personal experiences caring for older adults with dementia in Lithuania. Int J Ment Health Syst. 2021;15(1):12. 10.1186/s13033-020-00428-w. 33472676 10.1186/s13033-020-00428-w PMC7816390

[77] OECD. Health at a glance 2023: OECD indicators. Paris: OECD Publishing; 2023. 10.1787/7a7afb35-en.

[78] Agyemang-Duah W, Abdullah A, Rosenberg MW. Caregiver burden and health-related quality of life: a study of informal caregivers of older adults in Ghana. J Health Popul Nutr. 2024;43(1):31. 10.1186/s41043-024-00509-3. 38383532 10.1186/s41043-024-00509-3 PMC10882722

[79] Shoukr EMM, Mohamed AAER, El-Ashry AM, Mohsen HA. Effect of psychological first aid program on stress level and psychological well-being among caregivers of older adults with alzheimer’s disease. BMC Nurs. 2022;21(1):49. 10.1186/s12912-022-01049-z. 36217138 10.1186/s12912-022-01049-z PMC9551605

[80] Hu X, Liu H, Zhang L, Wang Y, Li Y. Mediating effect of social support on the relationships between caregiver burden and quality of life in family caregivers of people with dementia: a cross-sectional study in rural China. BMC Nurs. 2025;24:37. 10.1186/s12912-024-02671-9. 39794759 10.1186/s12912-024-02671-9 PMC11721535

[81] Jiang Y, Chen H, Lin C, Wang X. Relationship between anxiety and fatigue in dementia family caregivers: hope as a mediator. BMC Nurs. 2025;24:219. 10.1186/s12912-025-02853-z. 40011920 10.1186/s12912-025-02853-z PMC11863592

[82] Kim B, Kim JI, Na HR, Lee KS, Chae KH, Kim S. Factors influencing caregiver burden by dementia severity based on an online database from Seoul dementia management project in Korea. BMC Geriatr. 2021;21(1):649. 10.1186/s12877-021-02613-z. 34798814 10.1186/s12877-021-02613-z PMC8603539

[83] Ohno S, Chen Y, Sakamaki H, Matsumaru N, Yoshino M, Tsukamoto K. Burden of caring for alzheimer’s disease or dementia patients in Japan, the US, and EU: results from the National health and wellness survey: a cross-sectional survey. J Med Econ. 2021;24(1):266–78. 10.1080/13696998.2021.1880801. 33538195 10.1080/13696998.2021.1880801

[84] Shim M, Cho MJ, Lee JY, et al. Caregiving, care burden and awareness of caregivers and patients with dementia in Asian locations: a secondary analysis. BMC Geriatr. 2021;21:230. 10.1186/s12877-021-02178-x. 33827446 10.1186/s12877-021-02178-x PMC8028783

[85] Wang S, Qin J, Cheung DSK, et al. E-bibliotherapy for improving the psychological well-being of informal caregivers of people with dementia: a randomized controlled trial protocol. BMC Nurs. 2024;23:84. 10.1186/s12912-024-01706-5. 38303009 10.1186/s12912-024-01706-5 PMC10832133

[86] Leggins B, Hart DM, Jackson AJ, et al. Perceptions about Dementia clinical trials among underrepresented populations: a nationally representative survey of U.S. Dementia caregivers. Alz Res Ther. 2024;16:224. 10.1186/s13195-024-01579-5. 10.1186/s13195-024-01579-5 PMC11476697 39407319

[87] Ferré-Grau C, Raigal-Aran L, Lorca-Cabrera J, Lluch-Canut T, Ferré-Bergadà M, Lleixà-Fortuño M, et al. A mobile app-based intervention program for nonprofessional caregivers to promote positive mental health: randomized controlled trial. JMIR Mhealth Uhealth. 2021;9(1):e21708. 10.2196/21708. 33480852 10.2196/21708 PMC7864775

[88] Cho J, Ory MG, Stevens AB. Socioecological factors and positive aspects of caregiving: findings from the REACH II intervention. Aging Ment Health. 2016;20(11):1190–201. 10.1080/13607863.2015.1068739. 26213337 10.1080/13607863.2015.1068739

[89] Sorayyanezhad A, Nikpeyma N, Nazari S, Sharifi F, Sarkhani N. The relationship of caregiver strain with resilience and hardiness in family caregivers of older adults with chronic disease: a cross-sectional study. BMC Nurs. 2022;21(1):66. 10.1186/s12912-022-00966-3. 35821036 10.1186/s12912-022-00966-3 PMC9277877

[90] Xu L, Liu Y, He H, Fields NL, Ivey DL, Kan C. Caregiving intensity and caregiver burden among caregivers of people with dementia: the moderating roles of social support. Arch Gerontol Geriatr. 2021;94:104334. 10.1016/j.archger.2020.104334. 33516077 10.1016/j.archger.2020.104334

[91] Kaddour L, Kishita N. Anxiety in informal dementia carers: a meta-analysis of prevalence. J Geriatr Psychiatry Neurol. 2020;33(3):161–72. 10.1177/0891988719868313. 31409196 10.1177/0891988719868313

[92] Eplov LF, Petersen J, Jørgensen T, Johansen C, Birket-Smith M, Lyngberg AC, Mortensen EL. The mental vulnerability questionnaire: a psychometric evaluation. Scand J Psychol. 2010;51(6):548–54. 10.1111/j.1467-9450.2010.00834.x. 20642738 10.1111/j.1467-9450.2010.00834.x

[93] Jia X, Zhang Y, Li J, Wang J. The impact of caregiver burden on sense of coherence in Chinese family caregivers of people with dementia: the mediating effect of family resilience. BMC Psychol. 2025. 10.1186/s40359-025-02678-0 10.1186/s40359-025-02678-0 PMC11987476 40217520

[94] Nasreen S, Lim CJ, Kamil N, Razali R, Ibrahim NM, Tan MP, et al. Caregiver burden, mental health, quality of life and self-efficacy of family caregivers of persons with dementia in malaysia: baseline results of a psychoeducational intervention study. BMC Geriatr. 2024;24:656. 10.1186/s12877-024-05221-9. 39103767 10.1186/s12877-024-05221-9 PMC11301828

[95] Quinn C, Toms G. Influence of positive aspects of dementia caregiving on caregivers’ well-being: a systematic review. Gerontologist. 2019;59(5):e584–96. 10.1093/geront/gny168. 30597058 10.1093/geront/gny168

[96] Bressan V, Visintini C, Palese A. What do family caregivers of people with dementia need? A mixed-method systematic review. Health Soc Care Community. 2020;28(6):1942–60. 10.1111/hsc.13048. 32542963 10.1111/hsc.13048

